# Ziconotide-Induced Oro-lingual Dyskinesia: 3 Cases

**DOI:** 10.5334/tohm.431

**Published:** 2020-10-06

**Authors:** Kristopher Grajny, Jennifer Durphy, Octavian Adam, Sharmeen Azher, Megan Gupta, Eric Molho

**Affiliations:** 1 Parkinson’s Disease and Movement Disorders Center, Albany Medical Center, Albany, NY, US

**Keywords:** Ziconotide, Dyskinesia, Dysesthesias, Oral, Lingual

## Abstract

**Background:**

Ziconotide (ZCN), a nonopioid analgesic, is first-line intrathecal therapy for patients with severe chronic pain refractory to other management options. We describe three cases of ZCN-induced movement disorders.

**Cases:**

Case one is a 64-year-old woman who presented with oro-lingual (OL) dyskinesia with dysesthesias and bilateral upper extremity kinetic tremor. Case two is a 43-year-old man with a 20-month history of ZCN treatment who developed OL dyskinesia with dysesthesias, involuntary left hand and neck movements, hallucinations, dysesthesias on his feet, and gait imbalance. Case three is a 70-year-old man with a 4-month history of ZCN use who developed OL dyskinesia with dysesthesias.

**Conclusions:**

Intrathecal treatment of pain with ZCN may be complicated by a drug-induced movement disorder where OL dyskinesia is characteristic. The movement disorder is likely to be dose related and reversible with ZCN discontinuation, but a chronic movement disorder is also possible.

## Introduction

Ziconotide, a nonopioid analgesic approved in 2004, is an uncommon, non-opioid intrathecal therapy for patients with severe chronic pain refractory to other management options. ZCN inhibits nociceptive signal transmission in the spinal cord by selectively blocking presynaptic N-type voltage sensitive calcium channels of the dorsal horn. The drug is delivered via an intrathecal infusion pump. Side effects are dose related and ZCN labelling features a black box warning for severe neuropsychiatric and cognitive adverse events, which have been reported even in patients with no prior history of psychiatric disease [1,2,3,4,5,6]. Although “dyskinesia” is mentioned in a clinical trial report for the drug, almost no literature exists on ZCN-induced movement disorders [7].

## Case 1

A 64-year-old woman with chronic back pain following a resection of spine hemangioblastoma 20 years prior, presented with facial movements. In 2017 she had a ZCN pump placed, titrating to a maximum dose of 6 mcg per day. Her dyskinesias started in November 2018 and she first presented to our clinic in December 2018. Her symptoms consisted of involuntary movements of the lower face and tongue, accompanied by oral dysesthesias described as “a small mouse walks inside my mouth, with pointy toes that is stiff and very painful.” The movements were continuous while awake, and intermittently interfered with swallowing and chewing. There was no history of exposure to dopamine blocking agents such as anti-emetics or antipsychotics.

On exam, she had continuous, irregular movements of the lower face at rest with lip pursing and retraction, mild lateral deviations of the jaw, undulation of the upper throat and excessive eye blinking. With the mouth open there are writhing movements of the tongue. The movements occur during speech and interfere to a mild degree (see Video). A bilateral upper extremity kinetic tremor is also seen.

**Video:** **Case One is 64-year-old woman with stereotypic tongue and mouth movements.** Excessive eye blinking and upper limb action tremor is also seen.

Basic metabolic panel, liver functions, ceruloplasmin, B12, antiphospholipid antibodies, and serum paraneoplastic panel were normal. Her symptoms progressed and she developed delirium with auditory hallucinations. Her ZCN pump was decreased to a dose of 0.25 mcg and her symptoms improved but did not resolve completely. During the summer of 2019, she again developed worsening auditory hallucinations on this lower dose, so the pump was discontinued with gradual resolution of all neurological symptoms.

## Case 2

A 43-year-old man with type 1 diabetes, peripheral neuropathy, peripheral vascular disease, scleroderma, and psoriatic arthritis presented for evaluation of abnormal movements. He had a ZCN pump placed in March 2017 for painful diabetic neuropathy with an excellent response. In September 2018, his pump rate was increased to 7 mcg per day and then further increased in December 2018 to 9 mcg. His symptoms began in November 2018, when he noticed that his tongue was moving around constantly. He would roll his tongue over his teeth, stating it felt like “there was something in his mouth that he needed to clear out.” In January 2019, he developed involuntary movements of the wrists, and he began having motions of his head that his wife describes as “throwing his head back.” In addition, he reported a sensation as if there were “saran-wrap around his feet” and he would move his legs in bed to relieve the sensation. Treatment with trihexyphenidyl, quetiapine and ziprasidone (all started after symptom onset) provided no benefit. Clonidine 0.2 mg daily was somewhat beneficial for dysesthesias.

We first evaluated him in February 2019. He noted difficulties talking, chewing and swallowing. His movements were more prominent during stress, excitement, or fatigue. He felt he could not suppress them and there was no associated urge. On exam, his speech was dysarthric. He had constant, writhing movements of his tongue within his mouth. He had limited mouth opening and was unable to relax his tongue to the bottom of his mouth. He had intermittent sustained jaw opening and intermittent involuntary movements of the left arm which involved flexion at the elbow and elevation over his head. They would occur intermittently lasting for a couple of seconds. The movements were partially and temporarily attenuated with motor tasks in the limbs and would worsen during speech. Lab testing was normal including serum ceruloplasmin, T4, TSH, B12, ammonia, Lyme C6 ELISA and illicit drug screen.

His ZCN infusion was discontinued in March 2019. The oral dyskinesia and left arm movements gradually resolved over a period of several months. However, he continued to experience dysesthesias in the lower extremities chronically possibly due to concomitant neuropathy.

## Case 3

A 70-year-old man with cervical spondylosis, chronic cervicalgia and depression was referred for evaluation of involuntary mouth movements. For his neck pain, he had an intrathecal ZCN pump placed in August 2015. In late 2015, he began experiencing hallucinations and disorientation. His ZCN pump was at 2.4 mcg at the time. The ZCN dosage was decreased and these symptoms gradually improved. He first noticed tongue movements starting December 2015. He has a history of lichen planus in the mouth, and he believed that he started using his tongue to soothe the discomfort of the oral lesions. He felt like this was purposeful, but he eventually realized the movements were involuntary. They became more persistent and he had less control over them. He could briefly suppress the movements early in the day, but not later in the day. There was no specific urge associated with the movements and no relief. He also reported tongue sensation of “vibration” or “electricity.” At the patient’s request in August 2016, his ZCN pump was increased to 3.6 mcg to better control his pain. His medications were notable for Keppra 250 mg twice daily and Trileptal 300 mg twice daily as mood stabilizers, and Nortriptyline 25 mg for depression. There had been no exposure to dopamine receptor blocking drugs other than briefly to prochlorperazine several years prior.

He was evaluated in December 2016 in our movement disorders clinic. On exam, there were stereotypical mouth and tongue movements, which were irregular, arrhythmic, and continuous. He was able to partially suppress these movements. With distracting maneuvers, they were attenuated. There were no involuntary movements of the upper face or limbs. There were no signs of parkinsonism. TSH, B12, Lyme, and serum ceruloplasmin was normal. By March 2017, ZCN had to be stopped due to recurrent hallucinations. The dyskinesia completely resolved within a few weeks.

## Discussion

Hallucinations with ZCN infusions have been well described, but there are very few reports of an associated movement disorder. In an early case report of a single participant in an open label study, transient tongue movement and “nervousness” were felt to be related to a rapid increase in the dose [8]. A more recent case report describes head and upper limb dyskinesia in a pediatric cerebral palsy patient receiving both ZCN and baclofen [9]. We highlight three cases of oral dyskinesia associated with ZCN infusions (See Table 1). While N-type voltage-gated calcium channels are not directly implicated with dyskinesia, there is CNS penetration of ZCN, and off-target impacts are possible. It has also been shown that voltage-gated calcium channels can influence dopaminergic transmission. Specifically, D2 upregulation can result from enhanced calcium channel modulation [10]. Therefore, this indirect effect on dopamine transmission might account for both the hallucinations and dyskinesia observed with ZCN use.

**Table 1:** Comparison of Three Cases of Ziconotide-Induced Oropharyngeal Dyskinesias.

	Case 1	Case 2	Case 3

Age	64	43	70
Gender	Female	Male	Male
Medical conditions	Spinal hemangioblastoma (resected)	DM1, Peripheral neuropathy, scleroderma, psoriatic arthritis	Cervical spondylosis, depression
Centrally acting medications	duloxetine 30 mg AM and 60 mg PM, pregabalin 100 mg TID	cyclobenzaprine 10 mg, hydroxyzine 50 mg, clonazepam 2 mg PRN, topiramate 100 mg BID, vortioxetine 20 mg	levetiracetam 500 mg BID, pregabalin 50 mg QHS, nortriptyline 25 mg QHS, oxycodone 10 mg PRN
Movement disorder	Stereotypic tongue movements, lower facial twitching with tactile dysesthesias	Stereotypic tongue movements with tactile dysesthesias	Stereotypic tongue movements with tactile dysesthesias
Other neurological features	Delayed saccades, bilateral upper extremity kinetic tremor, dysphagia	Involuntary left hand and neck movements, hallucinations, tactile dysesthesias on feet, gait imbalance	N/A
Maximum ZCN dose	6 mcg	9 mcg	3.6 mcg
Duration of ZCN treatment at onset of movement disorder.	12 months	20 months	4 months
Outcome of ZCN discontinuation.	Mild residual dyskinesia	Complete resolution of movements	Complete resolution of movements

Legend: ZCN = Ziconotide, QHS = nightly, PRN = as needed, BID = twice daily, TID = three times daily.

Furthermore, all three cases presented with dysesthesias in the mouth associated with these movements. This observation mirrors the case series of oral and genital pain in tardive syndromes reported by Ford et al. and attests to the complex sensorimotor interactions within the PNS and CNS that are evident in tardive dyskinesia and other movement disorders [11]. Additionally, two of the cases reported brief distractibility (Cases two and three) along with some ability to suppress the movements (case three). While distractibility might suggest a functional disorder, there were no other functional exam findings and suppressibility is well known to occur in neuroleptic-induced tardive dyskinesia [12].

Evidence for a causal relationship between intrathecal ZCN and the movement disorder reported here include the temporal association with increased doses of ZCN and the near or complete resolution with ZCN discontinuation. In addition, the cases share a similar anatomic location and a movement disorder phenomenology most consistent with stereotypy. It is also of interest that in all three of our cases, an unpleasant complex oral sensation accompanied the movement disorder, perhaps related to ZCN’s activity at sensory pathways in the spinal cord and sensory integration areas of the brain or off-target interaction of ZCN with dopaminergic pathways.

One limitation of this case series is that they were analyzed retrospectively as the association between ZCN and involuntary movements was not immediately apparent. Also, the work up for other causes of the movement disorder was not uniform primarily because resolution of the movements with ZCN discontinuation obviated the need for additional diagnostic testing. These cases further suggest that this complication is dose related and may be related to rapid dose increases. Most cases thus far have also been transient, however in one of our cases, the movements did not completely resolve with drug discontinuation raising the possibility that a chronic movement disorder may also be a risk with ZCN.
